# Advances in Biotechnology for Sustainable Development

**DOI:** 10.1155/2016/7154916

**Published:** 2016-03-27

**Authors:** Weiqi Fu, Basel Khraiwesh, Hongbing Liu, Lei Kai

**Affiliations:** ^1^Division of Science and Math and Center for Genomics and Systems Biology (CGSB), New York University Abu Dhabi, P.O. Box 129188, Abu Dhabi, UAE; ^2^Center for Systems Biology and Faculty of Industrial Engineering, Mechanical Engineering and Computer Science, School of Engineering and Natural Sciences, University of Iceland, 101 Reykjavik, Iceland; ^3^National Institute of Diabetes and Digestive and Kidney Diseases (NIDDK), National Institutes of Health (NIH), 9000 Rockville Pike, MD 20892, USA; ^4^Max Planck Institute of Biochemistry, 82152 Martinsried, Germany

It may be mentioned that transformation towards a biobased industry is one of the big challenges facing our human society. Biotechnology is such a driver for change from petroleum-based economy to a more sustainable biobased economy. The use and development of biotechnologies for bioproduction and bioremediation are of great interest across a wide range of different communities. Moreover, during the past decades, development of tools in manipulating genetic framework empowered the cell factories as a reliable bioresource of recombinant protein production. The number of organisms that can be used as expression system is expanding.* Escherichia coli* was the most studied and used platform organism, then followed by the implementation of yeast as a eukaryotic model organism. Recently, algal cells, insect cells, and mammalian cells are catching up with their abilities of posttranslational modifications. In addition to the living cells,* in vitro* protein production is also emerging as one important tool for recombinant protein production, especially in membrane proteins, toxins, and aggregation prone proteins. Using cell lysate or purified proteins as the source of transcription and translation machinery, corresponding recombinant proteins of certain genes can be produced within several hours. The boosts of recombinant protein production develop along with the biophysics methods. Nuclear magnetic resonance (NMR) and liquid chromatography-mass spectrometry (LC-MS) are now routinely used as identification and characterization of recombinant proteins. Though the topics and papers selected here are not a fully comprehensive representation of the research area of biotechnology for sustainable development, we believe that the rich knowledge presented in these quality papers should be shared with the scientific community in the related fields.

This special issue received a submission of twenty-three manuscripts with an acceptance rate of approximately 22%. The specific papers are described with details as below.

The paper authored by R. Elkacmi et al. developed a quick and easy separation process for extracting oleic acid with high purity, biodegradable soap with a very good quality as well as glycerol from olive mill wastewater, which could cause detrimental effects on nature. It could recover valuable products with an industrial scale. However, so much waste going into the environment is neither reusable nor recyclable, so phytoremediation remains one of the best choices to clean up our environment.

The paper authored by H. Mubarak et al. presented an interesting work on the metabolic modifications in the species* Boehmeria nivea* L. subjected to different levels of the arsenic (As) concentration under hydroponic conditions. The cellular basis of the As tolerance in* B. nivea* L. is well explained. We should believe that, with emerging technologies designed specifically for protecting the environment, the globe will become clean and clear gradually again.

The paper authored by X. Zheng and D. Li studied the synergistic effects between* Rhizobium phaseoli* and* Acidithiobacillus ferrooxidans* in the bioleaching process of copper. The work presented demonstrated that mixed cultures using high-tolerance chemoheterotrophic bacteria achieved better performance than pure cultures in bioleaching process and suggested a method to address the challenge of low leaching rate and inefficiency.

The paper authored by I. Beltrán-Hernández et al. presented a study on removing cadmium from aqueous solutions using a natural adsorbent like nopal (*Opuntia albicarpa* L. Scheinvar). The results presented suggested that using the thermally treated nopal (TN) achieved the best removal of cadmium (53.3%, corresponding to 0.155 mg g^−1^) at pH 4.0.

The paper authored by Y. Ma et al. developed a colloidal gold immunochromatographic assay (GICA) for detecting immunodeficiency virus type 1 (HIV-1) p24 protein using mouse monoclonal antibodies (mAbs). The work presented achieved an overall specificity of 98.03% in the test and suggested that this method could be used as a convenient and efficient tool for early diagnosis of HIV infection.

